# Bovine tuberculosis source attribution using decision tree analysis: breakdown investigations in Italy (2022–2023)

**DOI:** 10.3389/fvets.2025.1609526

**Published:** 2025-08-13

**Authors:** Marco Tamba, Giorgio Galletti, Daniela Loda, Sara Salvato, Marco De Nardi, Maria Beatrice Boniotti

**Affiliations:** ^1^Epidemiology Unit, Istituto Zooprofilattico Sperimentale della Lombardia e dell’Emilia Romagna “B. Ubertini”, Bologna, Italy; ^2^National Reference Laboratory for Bovine Tuberculosis, Department of Animal Health, Istituto Zooprofilattico Sperimentale della Lombardia e dell’Emilia Romagna “B. Ubertini”, Brescia, Italy; ^3^Department of Veterinary Medical Sciences (DIMEVET), Alma Mater Studiorum – University of Bologna, Ozzano dell'Emilia, Italy

**Keywords:** bovine tuberculosis, breakdown, epidemiological investigation, *Mycobacterium tuberculosis* complex, risk factors

## Abstract

In this study, we present an overview of 348 Bovine Tuberculosis (bTB) breakdowns reported in Italy between January 2022 and December 2023, and declared resolved between January 2022 and February 2025. The main objective of this study is to investigate the most probable sources of these bTB breakdowns using decision tree analysis, and to compare the findings with conclusions drawn by official veterinarians. Most of the studied breakdowns (332; 95.4%) involved cattle herds only, 11 (3.1%) involved water buffalo herds only, and five (1.4%) involved multiple species. bTB was primarily detected in beef herds (82.8%), while mixed and dairy herds represented 10.3 and 6.9% of the breakdowns, respectively. In half of the breakdowns, the number of reactors was four or fewer. We also collected genotype data for 268 *Mycobacterium tuberculosis* complex isolates from 191 (54.9%) different breakdowns. *M. bovis* (255 isolates; 95.1%) came from 180 (94.2%) breakdowns, showing wide genetic variability. *M. caprae* (13 isolates; 4.9%) came from 11 (5.8%) breakdowns. Finally, we investigated the probable sources of infection, considering the five most frequently proposed sources of bTB breakdowns: (i) residual infection; (ii) introduction of infected cattle from other herds; (iii) sharing of pastures with infected herds; (iv) contiguous spread from infected neighboring herds; and (v) interaction with wildlife reservoirs. For each source, a decision tree was developed, and a likelihood of infection was assigned to each end node of the trees. The analysis identified residual infection (11.2%) as the most frequent source of bTB breakdowns, followed by sharing of pastures (10.9%) and interaction with wildlife (7.2%). The introduction of infected cattle and contiguous spread from infected neighboring herds were identified as less relevant sources. These tools allowed us to identify a likely source of infection in about 26% of cases. The results of our study, although based on scientific criteria, showed poor agreement with the conclusions of the veterinary officers who conducted the breakdown investigations in the field. In our opinion, these tools, when added to the “classic” investigation methodologies, should improve their effectiveness in identifying sources of infection in bTB breakdowns in Italy, supporting the eradication of this zoonotic disease.

## 1 Introduction

Bovine tuberculosis (bTB) is a chronic infectious disease affecting cattle, including all *Bos*, *Bubalus*, and *Bison* species. It is caused by any of the mycobacterial species within the *Mycobacterium tuberculosis*-complex (MTBC) (1). In high-income countries, bTB control programs primarily rely on routine intra-dermal skin tests and the removal of reactors, supplemented by slaughterhouse surveillance and control of animal movements (2, 3).

According to the European Union (EU) Animal Health Law (Regulation (EU) 2016/429), bTB eradication is mandatory in EU, and some countries have achieved disease-free status (DFS). This status requires reporting an annual herd-level incidence not exceeding 0.1% and maintaining at least 99.8% of bTB-free herds, covering at least 99.9% of the bovine population, over three consecutive years (Delegated Regulation (EU) 2020/689). However, despite intensive eradication efforts, bTB persists in some European countries, including several provinces of central and southern Italy (4).

**Figure 1:**
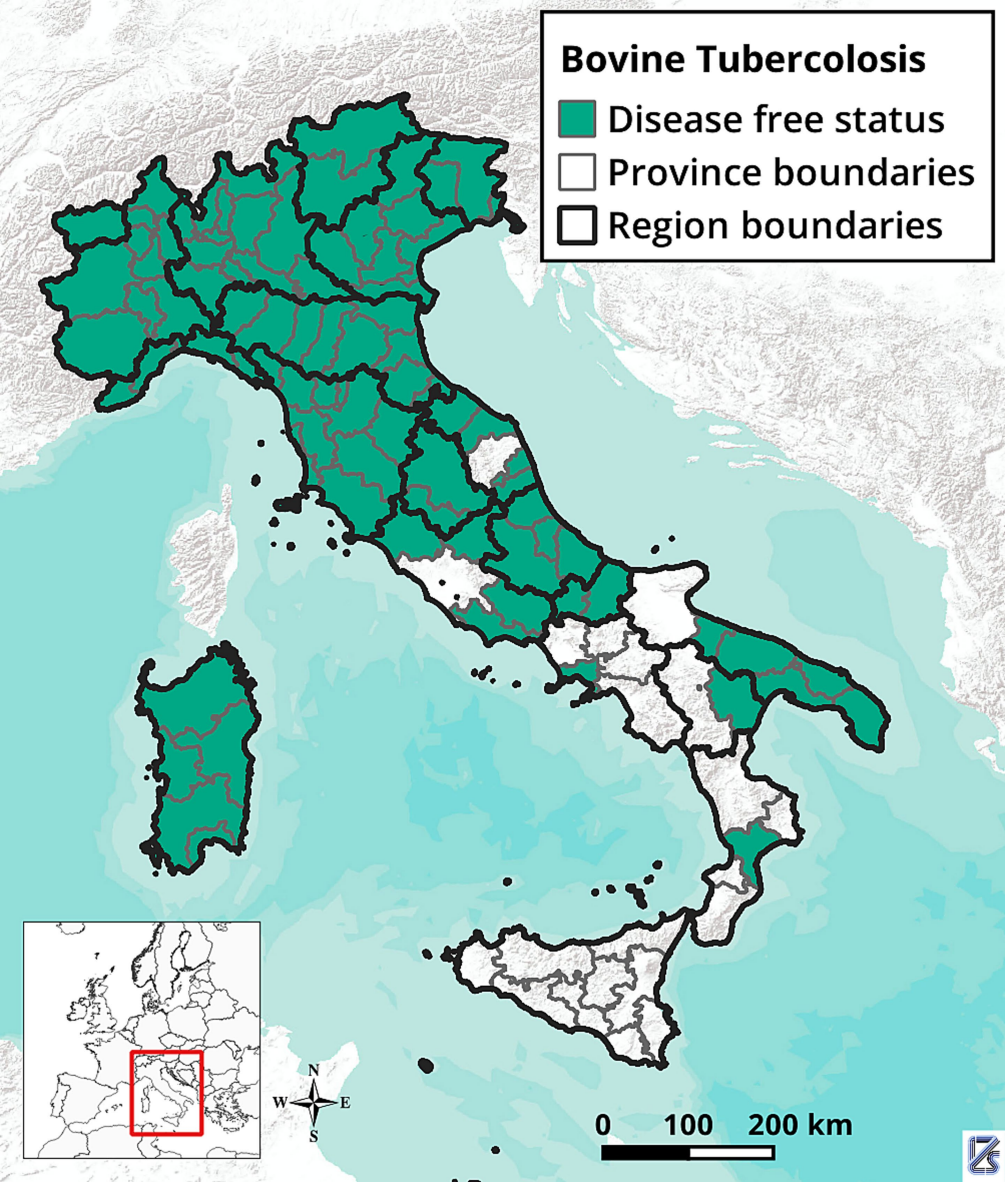
Map of Bovine Tuberculosis free provinces. Italy, 2024.

In Italy, the bTB control and eradication program began in 1995 and has been progressively strengthened over the years. However, DFS has not yet been achieved nationwide. Currently, 13 regions and 16 provinces have DFS, while one region (Sicily) and 12 provinces, mostly in Southern Italy (Figure 1), are still under the national eradication program (Implementing Regulation (EU) 2021/620). In 2023, the overall bTB herd prevalence (PR) and incidence rate (IR) were approximately 0.21 and 0.17%, respectively, with significant differences between DFS zones (PR: 0.04%; IR: 0.03%) and non-DFS zones (PR: 0.63%; IR: 0.52%). Since 2021, there has been a moderate increase of PR and IR (Statistics in the portal of the National Veterinary Information System^1^). This increase is related to both the higher number of new infected herds detected each year and the decrease in the number of cattle herds. Currently, MTBC infection has been virtually eradicated from housed dairy farms, while it is still present, particularly in beef cattle farms, in areas where grazing and transhumance are widely practiced. In these regions, the complexity of the disease epidemiology, traditional livestock management practices, and socio-cultural factors hinder the control of bovine tuberculosis.

The epidemiology of MTBC infection is characterized by its high resistance in the environment and wide host range (5). A bTB herd breakdown may occur due to the persistence of mycobacteria within the herd or its introduction into a previously free herd. Residual infection could result from false negatives in the skin test or suboptimal application of the test. Indirect transmission due to the microorganism’s hypothesized persistence in the environment could also contribute to residual infections (6–8). The infection of other mammal species, such as sheep, goats, and pigs on the farm, could contribute to the recirculation of MTBC within the cattle herd if these animals are not strictly segregated (5, 9).

External sources of bTB infection include the unwitting purchase of infected animals (8, 10, 11) and interaction with infected domestic mammals at common pastures (11, 12). The presence of neighboring bTB-infected herds may also introduce mycobacteria into a herd through direct contact with infected animals over farm boundaries or indirect contact, such as drainage of contaminated sewage (11, 12), exchanges of personnel, or contaminated equipment. In many countries, the presence of wildlife reservoirs endemically infected poses a challenge to bTB eradication schemes. Examples of such reservoirs include the European badger (*Meles meles*) in Great Britain and Ireland (8), red deer (*Cervus elaphus*) in Alpine region (13, 14), and wild boar (*Sus scrofa*) in Spain (15). In Italy, wild boar has been identified as the main bTB maintenance host (16, 17), although MTBC has also been isolated from other wildlife species, such as red deer, fallow deer (*Dama dama*), brown bear (*Ursus arctos marsicanus*), and porcupine (*Hystrix cristata*) (18–21).

Molecular genotyping of MTBC isolates in culture-confirmed cases has become an essential component of bTB epidemiological investigations, offering higher resolution in tracing transmission pathways than traditional field-based methods. Techniques such as spoligotyping, MIRU-VNTR, and, more recently, whole genome sequencing (WGS) allow for the discrimination of isolates circulating in different regions, the identification of transmission clusters at various geographical and evolutionary scales, and the distinction between reinfection and persistence within the same herd (22–24). In areas where bTB remains endemic, genotyping data, alongside epidemiological metadata, can help determine whether recurrent breakdowns are due to residual infection or new introductions, including those from wildlife reservoirs or neighboring herds (25). Furthermore, integrating molecular data with spatial and temporal information enables the reconstruction of transmission networks and the detection of potential interspecies spillover events (26). These insights are particularly valuable in complex epidemiological contexts, such as Southern Italy, where multiple sources and routes of infection may coexist (26). In Italy, genotyping of *M. bovis* and *M. caprae* isolates has been used since 2000 to support breakdown investigations (17, 22).

Determining the pathways by which herds become infected and quantifying their relative importance could provide valuable information for identifying the most appropriate and cost-effective preventive measures. Therefore, the main objective of this study was to identify the most likely sources of bTB breakdowns notified in Italy between 2022 and 2023.

## 2 Materials and methods

### 2.1 Data

The Italian national bTB eradication program, in accordance with Delegated Regulation (EU) 2020/689, is based on annual testing of cattle and the culling of bTB-infected cattle. Each year, all animals older than 6 weeks in every herd are tested using the single intradermal cervical tuberculin test (SICT). In DFS provinces, risk-based surveillance is carried out, and testing frequencies vary according to regional regulations. Additionally, all slaughtered cattle undergo post-mortem inspection by an official veterinarian, and all cattle older than 6 weeks are tested using the SICT within 30 days prior to being moved to another herd.

Test results are stored in a national database, named SANAN, which is maintained by the Italian Ministry of Health (MoH). Cattle herds are classified as bTB-free if no positive animals are detected in at least two consecutive follow-up herd tests. Herds with at least one SICT-positive animal are classified as suspect cases and are further investigated to confirm the presence of MTBC infection. Confirmation of infection is performed through post-mortem inspection and the sampling of organs and tissues for biomolecular assays (PCR) and culture to identify the causative agent (27). A bTB breakdown must be officially notified if any of the following criteria are met:

- MTBC is isolated; or
- at least one PCR-positive animal is detected along with tuberculous lesions or an epidemiological link to another bTB breakdown; or
- at least one SICT-positive animal is found with tuberculous lesions or an epidemiological link to another bTB breakdown.

If the herd is confirmed as infected, an epidemiological investigation is conducted by a veterinary officer, and the data are stored in a national database, named SIMAN, which is maintained by the MoH. The results of the inquiry are recorded in a questionnaire that includes data about herd management, the history of bTB testing results, animal movements, and interactions with other domestic and wild animals. Additionally, the veterinary officer (VO) conducting the inquiry records their own conclusions on the probable source of the breakdown. Since the VOs can modify the questionnaire in SIMAN until the breakdown is declared resolved, in our study we included only the outbreaks that occurred between January 2022 and December 2023 and were declared resolved following the reacquisition of the disease-free status between January 2022 and February 2025. The data were obtained from the MoH.

We also had access to the ear tag numbers of all animals involved in the breakdown from the date of suspicion to the date of breakdown resolution, which allowed us to trace individual animal movements. These data were obtained from the National Cattle Database (BDN) managed by the MoH, within the VETINFO portal, which also contains the SANAN and SIMAN databases. These data are usually available also to the VOs conducting the breakdown investigations.

Most Italian regions carry out a wildlife monitoring program for MTBC, primarily targeting deer and wild boar. Monitoring schemes vary locally, but typically involve the random sampling of head lymph nodes from hunted animals. Samples, regardless of the presence of lesions, are examined by culture. Some laboratories use PCR as a screening test and only culture PCR-positive samples. In Italy, all official laboratories involved in MTBC diagnosis are ISO/IEC 17025-accredited and follow the methods prescribed by the WOAH manual (1, 27).

Moreover, as part of the Italian bTB control and eradication program, all MTBC isolates from domestic and wild animals are submitted by the official laboratories to the National Reference Laboratory (NRL) for bTB, where they are archeived, and systematically genotyped using spoligotyping (28), Mycobacterial Interspersed Repetitive Unit-Variable-Number Tandem Repeats (MIRU-VNTR) with ETRA-E markers (29) and seven additional MIRU-VNTR markers selected by Boniotti et al. (22) to better discriminate the most common spoligotype isolate in Italy (SB0120). Spoligotype names were assigned according to the *Mycobacterium bovis* Spoligotype Database^2^ (30). The genotyping results are stored in a database, named ITAN-TB, which also contains data related to MTBC isolate records dating back to 2000 from different mammalian species, including humans. This database is maintained by the NRL located at the Istituto Zooprofilattico Sperimentale della Lombardia e dell’Emilia Romagna in Brescia.

### 2.2 Statistical analysis

Descriptive statistics were calculated for the number of breakdowns, number of infected animals and within-herd incidence, stratified by production type (i.e., beef, dairy, or mixed) and provincial status (DFS vs. non-DFS), based on 288 breakdowns recorded in the SIMAN database between 2022 and 2023. Group differences were assessed using the Kruskal-Wallis test, followed by Dunn’s post-hoc test for pairwise comparisons related specifically to the “type of production” variable. The Kruskal-Wallis test was employed as a non-parametric alternative to ANOVA. The variables analyzed included herd size, number of cases, and within-herd incidence. Statistical significance was defined as *p* < 0.05. All analyses were performed using the open-source software R (version 4.3.0) (31).

### 2.3 Investigation of the most likely source of bTB herd breakdowns

To assess the most likely source of bTB breakdowns, we followed these steps:

#### 2.3.1 Determination of possible sources of a bTB breakdown

Based on most common risk factors for bTB (8, 11, 32), we considered five possible sources of herd breakdowns: (i) residual infection; (ii) introduction of infected cattle from other herds; (iii) sharing pastures with infected herds; (iv) contiguous spread from infected neighboring herds; (v) interaction with wildlife reservoirs. If the origin of the breakdown could not be attributed to any of these sources, it was considered unknown.

#### 2.3.2 Determination of different events within each possible source through decision trees

**Figure 2:**
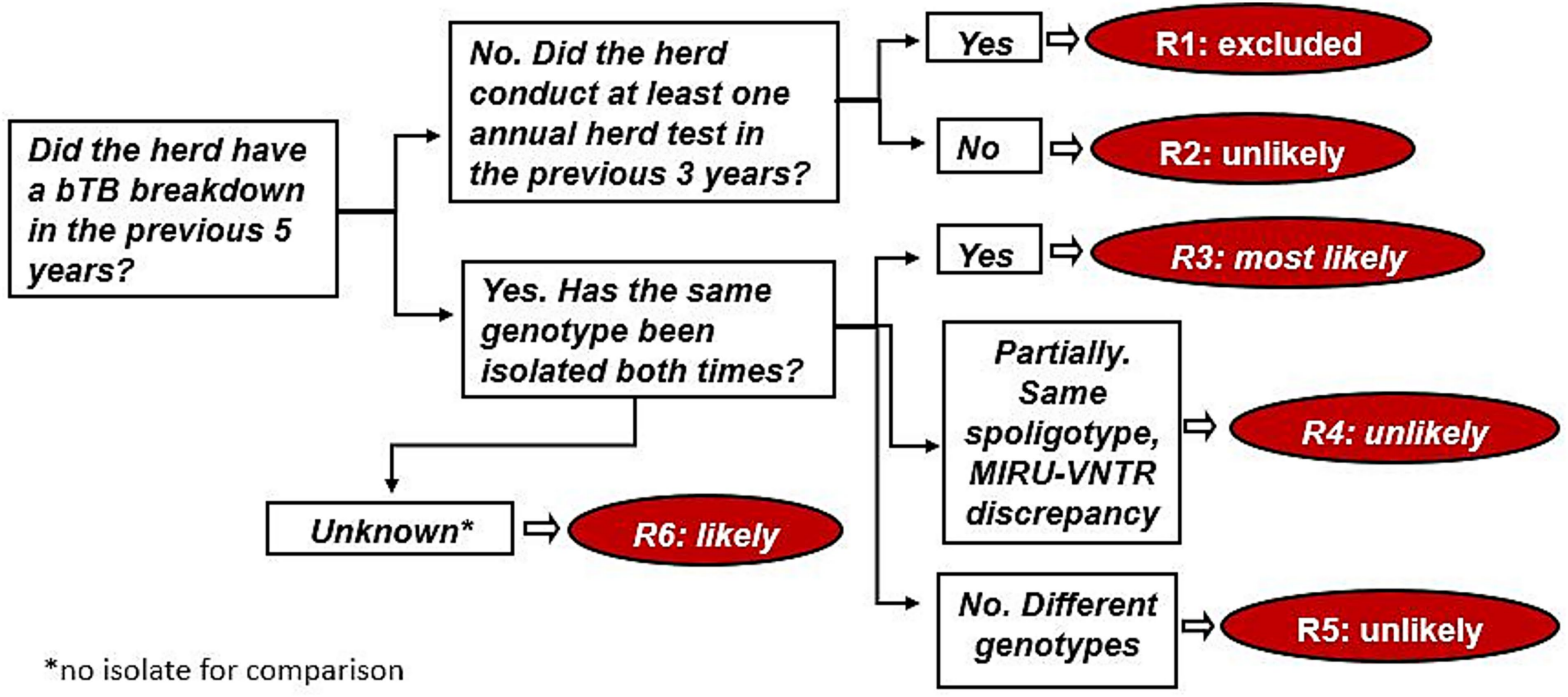
Decision tree diagram for residual infection.

Starting from the work of Guta et al. (32), decision trees were adapted to the available data. Five models representing a series of related events were designed for all the sources considered. Tree diagrams began with a single key question (root node), which branched into self-excluding occurrences (decision nodes); these, in turn, branched off into different possible events (end nodes). In Figure 2, the decision tree for residual infection is shown. For example, event R3 in Figure 2 corresponds to an infected herd that had a bTB breakdown in the previous 5 years and for which the same MTBC genotype was isolated both times. All five decision tree diagrams are included in the Supplementary Figures S1–S5.

In our study, two MTBC isolates were considered identical only if they had the same spoligotype and the same 12 MIRU-VNTR markers.

#### 2.3.3 Assessment of the likelihood of occurrence of different events

Based on our experience, end nodes were categorized into four qualitative risk classes: most likely (ML), likely (LI), unlikely (UN), and excluded (EX), representing the likelihood of MTBC entering the herd. In this work, we considered only events classified as “most likely” or “likely” as probable sources of the breakdown. Specifically, the categories “likely” and “most likely” approximately correspond to “weak epidemiological evidence” and “strong microbiological evidence,” respectively, based on the classification of the nature of evidence defined by EFSA (33) for the harmonized reporting of foodborne outbreaks across the EU.

#### 2.3.4 Data management and determination of different events that occurred in each breakdown

Based on available data for each breakdown, we determined the events within each possible source of infection, following the criteria described in the decision trees. For each breakdown, all five decision trees were applied in the same order (from S1 to S5), and the corresponding outputs were recorded.

These operations were performed by combining the data extracted from the SIMAN, SANAN, BDN, and ITAN-TB databases based on the time and space specified in each branch of the tree. Therefore, each breakdown ended with a likelihood of MTBC entry for each of the five investigated sources of breakdown. To perform this task, six MS-Access databases were developed: one for the raw data and one for each decision tree. Thanks to this approach, relevant data in the different data files were processed through source-specific queries, and a new dataset was generated containing the five likelihoods for the breakdowns included in the study.

#### 2.3.5 Determination of the most likely source of each bTB breakdown

To determine the most likely source of the breakdown for each infected herd, the likelihoods of the five different sources were compared following this criterion:

- In those breakdowns in which only one source (i.e., probability of MTBC entry ML or LI) was identified, we considered this option as the source of the entry of MTBC.
- In those breakdowns in which two possible sources were identified, we considered both options as equally probable.
- In those breakdowns in which three or more sources were identified (i.e., three or more values ML or LI), the source of infection was considered unknown.
- When all five possible sources of breakdown had values corresponding to UN or EX, the source of infection of the herd was considered unknown.

### 2.4 Most likely sources of breakdown attributed by veterinary officers versus sources obtained in our study

One of the questions that the veterinary officers had to complete in the epidemiological questionnaire was their opinion about the possible source of the breakdown. They had the option to provide more than one possible source within a closed list (Unknown, Residual infection, Introduction of infected animals, Pasture, Wildlife, Direct contacts, Indirect contacts). We considered direct and indirect contacts as proxies for contiguous spread. In those breakdowns in which two options had been provided, we considered these sources as equally likely. When the veterinary officers had selected more than two options, we considered the source of the breakdown as unknown. To evaluate the concordance between the opinion of veterinary officers and our results, we made the comparison only for those herds in which a single source of infection had been obtained by both methods. The agreement between both results was assessed by the Kappa value (34) by using the epiR library of the free software R version 4.3.0 (35). We interpreted the Kappa value as follows: values ≤ 0 as indicating no agreement and 0.01–0.20 as none to slight, 0.21–0.40 as fair, 0.41–0.60 as moderate, 0.61–0.80 as substantial, and 0.81–1.00 as almost perfect agreement (34).

## 3 Results

### 3.1 Descriptive results

**Figure 3:**
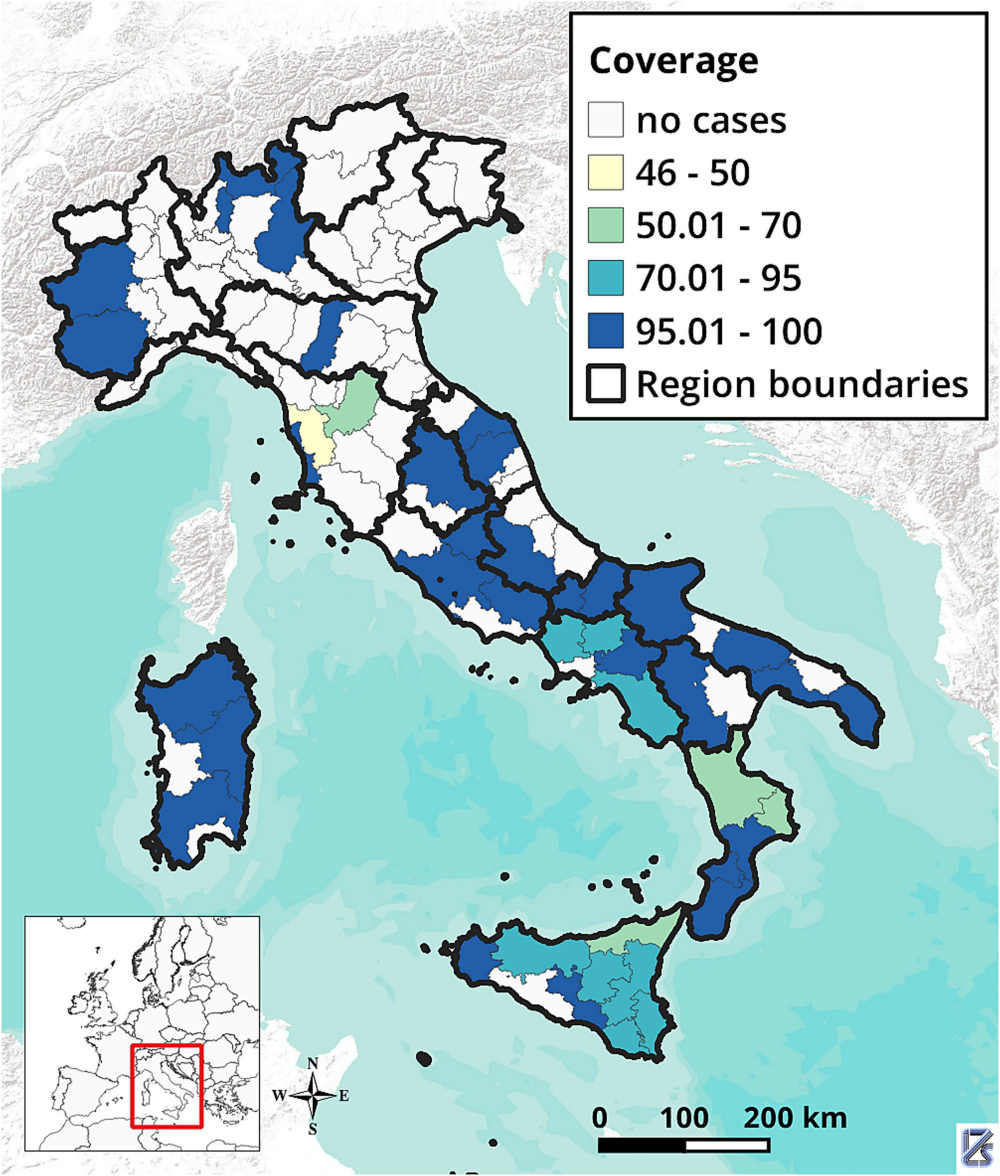
Percentage of Bovine Tuberculosis breakdowns covered by the study Italy, 2022–2023.

As of March 13, 2025, when data collection ended, information from 348 breakdowns declared resolved among those notified in 2022–2023 were collected. Figure 3 shows the geographical distribution of these breakdowns. Most (332; 95.4%) involved cattle herds only, 11 (3.1%) involved water buffalo (*Bubalus bubalis*) herds, and five involved multiple species: three (0.9%) included both cattle and buffaloes, and two (0.6%) included cattle and goats. These 348 breakdowns represented 86.1% of the 404 breakdowns reported in Italy between 2022 and 2023. Data were available for 44 breakdowns from regions with DFS and 304 breakdowns from non-DFS provinces, still under the eradication program. Descriptive statistics on herd size, number of infected animals and within-herd incidence by herd type (beef, dairy, or mixed) and management (housed, semi-open, transhumant) are reported in Table 1. The same statistics by herd type and disease status of the province are presented in Table 2. Bovine TB herd breakdowns were mostly detected in beef herds (82.8%), while mixed and dairy herds represented 10.3 and 6.9% of the breakdowns, respectively.

**Table 1:** Bovine Tuberculosis: median number and interquartile range of herd sizes, disease cases and within herd incidence (expressed as a percentage), by herd type and management, Italy, 2022–2023.

Herd type		Beef	Dairy	Mixed	Total
Management		Median (IQR)	Median (IQR)	Median (IQR)	Median (IQR)
Housed	N.breakdowns	130	23	20	173
	Herd size	41.5 (17–90)	85 (56–210.5)	32 (22.5–64.5)	46 (19–102)
	N.cases	2 (1–6.8)	9 (3–14)	10 (4.2–20)	3 (1–10)
	Herd incidence (%)	7.4 (2.9–23.3)	7 (3.5–15.2)	34.4 (8.3–50.4)	7.7 (3.3–30)
Semi-open	N.breakdowns	125	1	13	139
	herd size	44 (24–72)	10	30 (21–78)	43 (22.5–72.5)
	N.cases	5 (2–13)	1	9 (3–15)	5 (2–13.5)
	Herd incidence (%)	14.3 (5.6–40)	10	13.5 (7.7–42.9)	13.5 (5.9–40.6)
Transhumant	N.breakdowns	33		3	36
	Herd size	50 (27–90)		122 (77–123.5)	51 (28.5–104.8)
	N.cases	2 (1–7)		27 (18–35.5)	2 (1–8)
	Herd incidence (%)	6.1 (2.5–9.5)		28.1 (25.1–31.7)	6.3 (2.7–12.8)
Total	N.breakdowns	288	24	36	348
	Herd size	44 (20.8–79)	84.5 (52–209.2)	32.5 (21–84)	45 (21–82)
	N.cases	3 (1–9)	8.5 (2.8–13.5)	10 (3.8–20)	4 (1–11)
	Herd incidence (%)	9.3 (4–30.6)	7.1 (3.7–15)	28.3 (8.4–44.6)	9.6 (4.2–31.9)

IQR, interquartile range.

**Table 2:** Bovine Tuberculosis: median number and interquartile range of herd sizes, cases and within herd incidence (expressed as a percentage), by herd type and province disease status, Italy, 2022–2023.

Herd type		Beef	Dairy	Mixed	Total
Province status		Median (IQR)	Median (IQR)	Median (IQR)	Median (IQR)
DFS	N.breakdowns	37	3	4	44
	Herd size	43 (15–99)	52 (32.5–56)	51 (32.2–77.2)	45 (15–87.8)
	N.cases	1 (1–2)	4 (2.5–23)	4 (2.5–8.5)	1 (1–3.2)
	Herd incidence (%)	4.5 (1.2–16.7)	7.7 (7.2–44.2)	8.6 (6.2–12.2)	6.1 (1.3–16.7)
Non-DFS	N.breakdowns	251	21	32	304
	Herd size	44 (21–77)	108 (67–213)	31.5 (21–84.5)	45 (21.8–82)
	N.cases	3 (1–10)	9 (3–13)	11 (4.8–23)	4 (1–12)
	Herd incidence (%)	9.8 (4.4–31)	7 (3.2–14.8)	31.4 (8.8–50.4)	10.5 (4.5–32.9)

IQR, interquartile range; DFS, Disease Free Status.

In half of the breakdowns, the number of infected animals (cases) was four or fewer. Most of the cases were reactors, but the number also includes any SICT-negative animals that tested positive by PCR or culture. This value is largely influenced by figures from beef farms. The median herd size was significantly higher in dairy herds (84.5 animals), compared to beef (44 animals) and mixed herds (32.5; respectively *p* = 0.001 and *p* = 0.006). No differences were detected for herd size between DFS and non-DFS provinces. The median number of disease cases was significantly higher in mixed herds (10 cases) compared to beef herds (3 cases; Dunn’s test p = 0.001). The median within-herd incidence was significantly higher on breakdowns detected in mixed herds (28.3%; *p* = 0.01) compared to beef herds (9.3%) or dairy herds (7.1%). In non-DFS provinces, a significantly higher number of cases and within-herd incidence (Kruskal-Wallis test, *p* ≤ 0.01) was registered.

We collected genotype data for 268 MTBC isolates from 191 (54.9%) of the 348 bTB breakdowns. Multiple MTBC genotypes were identified in 43 herds, accounting for 22.5% of the culture-confirmed breakdowns. *Mycobacterium bovis* (255 isolates; 95.1%) came from 180 (94.2%) breakdowns. Twenty-six different spoligotypes of this mycobacterial species were detected, with SB0120 (48.2%), SB0841 (21.2%), and SB0134 (11.8%) being the most represented. One hundred and five different MIRU-VNTR profiles were identified, although for 15 isolates not all 12 markers were determined. *Mycobacterium caprae* (13 isolates; 4.9%) came from 11 (5.8%) breakdowns. Three different *M. caprae* spoligotypes were detected, with SB0418 (76.9%) being the most represented. Twelve different MIRU-VNTR profiles were identified, although for one isolate not all 12 markers were determined. No cases of *M. bovis/M. caprae* coinfection were detected in the studied herds.

### 3.2 Most likely source of breakdown based on decision trees

The most likely sources of herd breakdowns in Italy are shown in Table 3. The frequencies of the defined events for each source are reported in the Supplementary materials (Supplementary Tables S1–S5). Residual infection (11.2%; 95% CI: 8.3–15.0%), followed by sharing of pastures (10.9%; 95% CI: 8.1–14.6%), and interaction with wildlife reservoirs (7.2%; 95% CI: 4.9–10.4%) were identified as the most important sources. The introduction of infected cattle and contiguous spread from infected neighboring herds were identified as less relevant sources. In 258 herds (74.1%), the origin of infection remained unknown. In 24 of these (6.9%), more than one possible source was determined. If only herds with one or two sources were considered (Table 4), residual infection was again the most likely source, followed by sharing of pastures and introduction of infected cattle. In this case, the importance of contiguous spread was much lower. There were also differences in the sources of bTB breakdowns according to the location of the herd (Table 4). In DFS zones, residual infection and introduction of infected cattle showed higher proportions compared to non-DFS provinces, where sharing of pastures and interaction with wildlife reservoirs seemed to have higher importance. The proportion of bTB breakdowns with unknown sources is estimated at about 74%, with no differences due to the location of the farm. This high value was primarily due to the absence of MTBC molecular data for comparison, which were lacking for 157 (45.1%) of the 348 study cases. There were differences in the sources of bTB breakdowns according to the type of herd (Table 5). In dairy herds, residual infection was more relevant compared to beef herds, while wildlife, pasture, or contiguous spread seemed to have less importance. Residual infection was also relevant in mixed herds, while in beef herds, sharing of pastures seemed a significant source of MTBC infection compared to dairy and mixed herds.

**Table 3:** Probable sources of bovine tuberculosis breakdown identified using decision trees, Italy, 2022–2023.

Source of breakdown	No. of breakdowns		Total	Proportion (on 348 herds)	95% CI
	Most likely	Likely			
Residual infection	10	29	39	11.2%	8.3–15.0%
Introduction of infected cattle	14	5	19	5.5%	3.5–8.4%
Contiguous spread	12	2	14	4.0%	2.4–6.6%
Sharing of pastures	12	26	38	10.9%	8.1–14.6%
Interaction with wildlife	25	0	25	7.2%	4.9–10.4%
Unknown (a)			258	74.1%	69.3–78.5%
Total (b)	73	62	393		

(a) In 248 herds the likelihood of all the sources was “unlikely” or “excluded”; (b) In 24 herds there was more than one “likely” or “most likely” source of MTBC entry; 95% CI: 95% confidence interval.

**Table 4:** Frequency of bovine tuberculosis breakdowns sources identified using decision trees, by status of the province, Italy, 2022–2023.

Status of zone	DFS provinces		Non-DFS provinces		Total	
Source of breakdown	Obs (b)	%	Obs (b)	%	Obs (b)	%
Residual infection	5.5	12.5%	26	8.6%	31.5	9.1%
Introduction of infected cattle	3	6.8%	13	4.3%	16	4.6%
Contiguous spread	2	4.5%	4	1.3%	6	1.7%
Sharing of pastures	1.5	3.4%	22	7.2%	23.5	6.8%
Interaction with wildlife	0	0.0%	13	4.3%	13	3.7%
Unknown (a)	32	72.7%	226	74.3%	258	74.1%
Total	44	100.0%	304	100.0%	348	100.0%

Obs: number of studied breakdowns; (a) for 10 bTB breakdowns more than two “likely” or “most likely” sources were identified; (b) breakdown with two sources counted for 0.5.

**Table 5:** Frequency of sources of bovine tuberculosis breakdowns identified using decision trees, by herd type, Italy, 2022–2023.

Herd type	Beef		Dairy		Mixed		Total	
Source of breakdown	Obs (b)	%	Obs(b)	%	Obs(b)	%	Obs(b)	%
Residual infection	22.5	7.8%	5	20.8%	4	11.1%	31.5	9.1%
Introduction of infected cattle	12	4.2%	2	8.3%	2	5.6%	16	4.6%
Contiguous spread	5	1.7%		0.0%	1	2.8%	6	1.7%
Sharing of pastures	22.5	7.8%		0.0%	1	2.8%	23.5	6.8%
Interaction with wildlife	13	4.5%		0.0%		0.0%	13	3.7%
Unknown (a)	213	74.0%	17	70.8%	28	77.8%	258	74.1%
Total	288	100.0%	24	100.0%	36	100.0%	348	100.0%

Obs: number of studied breakdowns; (a) for 10 bTB breakdowns more than two “likely” or “most likely” sources were identified; (b) breakdown with two sources counted for 0.5.

Of the 191 bTB breakdowns with culture-confirmation, only in 122 (63.9%) cases was at least one additional MTBC isolate available for genotype comparison. When using genotype comparison alone to identify the source of infection, we observed a complete match in over 90% of cases for residual infection and pasture sharing. In contrast, a genetic correlation was detected in only about half of the cases involving the introduction of infected animals or contiguous spread. Although wildlife interaction was investigated in most cases, a full genetic match was found in only one quarter of them (Table 6).

**Table 6:** Probable sources of bovine tuberculosis breakdown identified using isolates comparison, Italy, 2022–2023.

Source of breakdown	No. of breakdowns with isolates for comparison	Proportion % (on 122 cases)	No. of breakdowns with full isolate match	Proportion %
Residual infection	11	9.0%	10	90.9%
Introduction of infected cattle	27	22.1%	15	55.6%
Contiguous spread	26	21.3%	14	53.8%
Sharing of pastures	40	32.8%	38	95.0%
Interaction with wildlife	92	75.4%	25	27.2%

Cases: number of bovine tuberculosis breakdowns with at least two MTBC isolates for comparison.

### 3.3 Results of our study versus conclusions from veterinary officers

**Figure 4:**
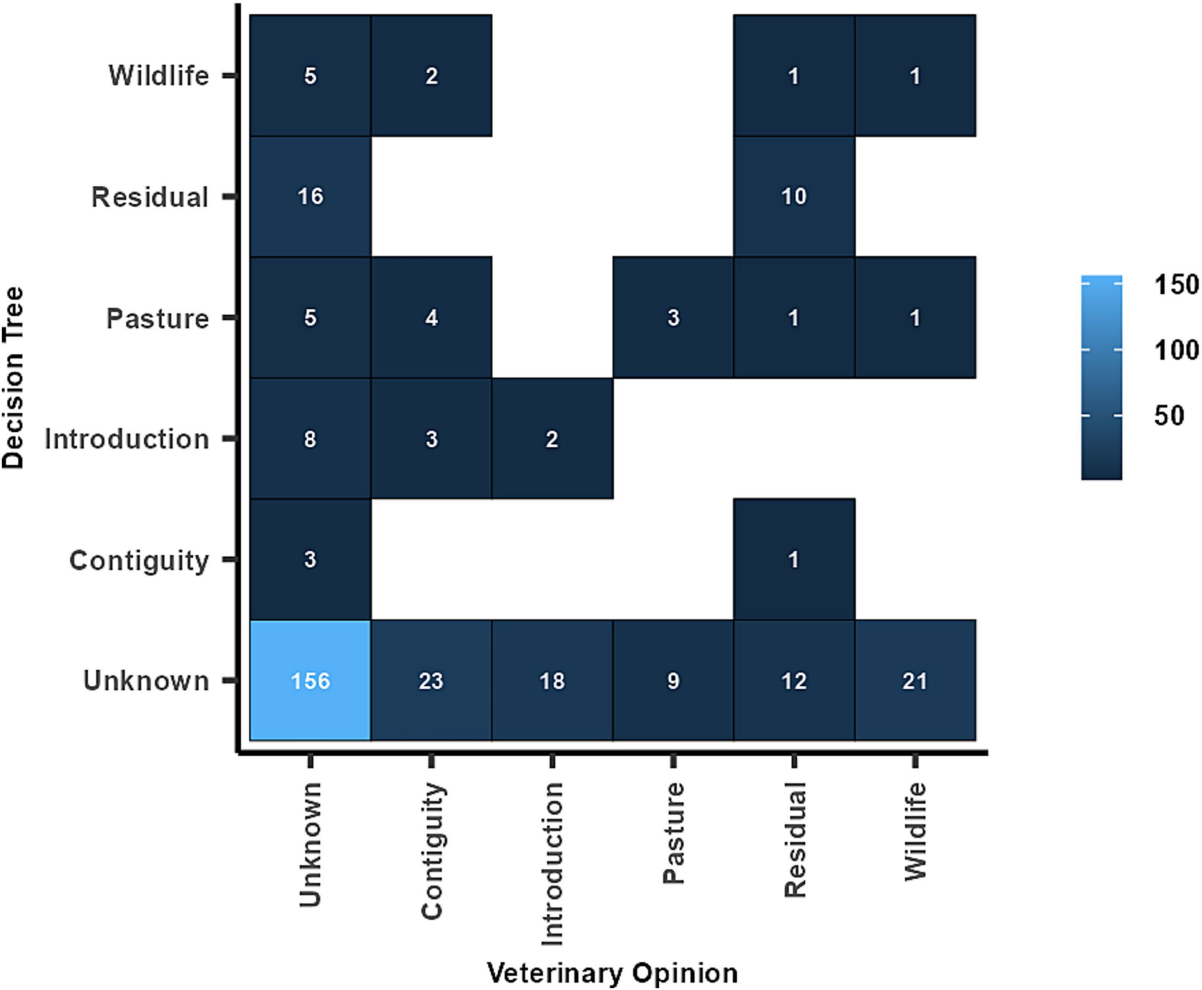
Agreement between sources of Bovine Tuberculosis breakdown where both our study and veterinary officers identified only one source.

For 305 breakdowns (87.6%) only one single source was identified by both the decision trees and the veterinary officers. The analysis revealed a poor agreement for all the identified sources of breakdowns (Table 7 and Figure 4); contiguous spread and interactions with wildlife had the worst agreement, with, respectively, zero and one breakdown in agreement.

**Table 7:** Agreement between sources of bovine tuberculosis breakdowns (breakdowns with single source) identified by decision trees and veterinary officers, Italy, 2022–2023.

Source of breakdown	Decision tree	Veterinary Officer	Agreement	Kappa	95% CI
Residual infection	26	25	10	0.33	0.22–0.45
Introduction of infected cattle	13	20	2	0.07	−0.04 – 0.18
Contiguous spread	4	32	0	−0.02	−0.09 – 0.04
Sharing of pastures	14	12	3	0.2	0.08–0.31
Interaction with wildlife	9	23	1	0.02	−0.08 – 0.12
Unknown	239	193	156	0.07	−0.03 – 0.18
Total	305	305	172		

95% CI, 95% confidence interval.

## 4 Discussion

The goal of breakdown investigations is to analyze disease breakdowns to gain control and prevent further spread of infection. A crucial step involves implementing measures to halt transmission, reduce new cases, and minimize the risk of future breakdowns (36). Prevention and control activities, through the application of appropriate diagnostic procedures, focus on identifying and controlling infection sources (tracing back) and verifying pathogen transmission to other herds (tracing forward). These measures should be integrated throughout the investigation to prevent any further cases. Several tools can be used to ensure a coordinated and well-organized investigation, among which questionnaires allow a systematic approach to gathering necessary information, including a standardized set of questions for farmers. Questionnaires play a key role in hypothesis generation and facilitate data collection from various sources (e.g., animal registers, laboratory results, field data) for both descriptive and analytic studies to identify the breakdown source (36). Due to these complexities, epidemiological investigations require skilled and specially trained personnel, as determining the source of infection, especially in chronic diseases, can be challenging.

The decision trees used in our study were designed based on those developed by Guta et al. (32) and were adapted to available data to investigate the five most frequent sources of bTB breakdown described in the literature (8, 11). In our study, we did not consider some potential sources of infection, such as transmission from infected humans or other domestic species like goats, pigs, or sheep. The ITAN-TB database has not recorded *M. tuberculosis* isolate from cattle in the last 25 years, while *M. bovis* has been sporadically identified in goats and sheep (26, 37–39). However, in Italy, data on the prevalence of MTBC infection in these species remain limited. Moreover, only two out of the 348 breakdowns considered involved herds with goats, despite their potential role in bTB epidemiology (9, 38, 40, 41). The role of pigs in bTB epidemiology has traditionally been considered minimal as they are mainly raised in intensive housed systems and slaughtered at young ages (9, 37). However, in central and southern Italian regions, there is a significant population of autochthonous pig breeds kept in free-range systems, sharing natural resources with both wild and domestic animals. In these areas, cases of *M. bovis* infection have been reported in pigs, often with generalized lesions (26, 42).

A key aspect of our decision trees is assigning a likelihood to each possible event. By applying this approach, we were able to assess different potential sources of bTB breakdowns based on available data and ideally identify the most likely source of infection. To strengthen the analysis, we used the molecular characterization of different MTBC isolates to provide stronger evidence of breakdown origin. In other studies, only spoligotyping was used for source attribution (32, 43), but this can lead to an overestimation of the extent of clustering (17). We believe that combining spoligotyping with MIRU-VNTR can lead to a more accurate identification or exclusion of a match. However, it is probable that also our study may have overestimated the likelihood of epidemiological linkage as a result of incomplete genotyping of the isolates. At the same time, it is possible that we excluded correlations between isolates from the same genetic cluster due to differences in only one MIRU-VNTR marker (17, 24). WGS is deemed the most appropriate technique for this purpose, as it allows for a comprehensive assessment of genome similarity and potential evolutionary relationships between two isolates (24). However, WGS is currently not universally applicable to all isolates in our country, and is primarily utilized in targeted research studies (17).

Unfortunately, molecular data were unavailable for 45% of breakdowns, limiting the ability to accurately assess the likelihood of specific sources. However, in approximately one third of the breakdowns investigated, it was possible to perform a genotype comparison, and the results largely confirmed those obtained through decision tree analysis. The absence of molecular data could be due to the lack of tissue sample collection at the abattoir, failure to recover mycobacteria by culture, or the presence of non-typable isolates (32). The absence of isolates for comparison, coupled with the short study period (2 years) and the limited number of cases–mainly because bTB outbreaks in Italy are fortunately less frequent than in other countries–has constrained the evaluation of the usefulness of decision trees for source attribution. Greater efforts should be made in the future to obtain at least one isolate from each bTB breakdown.

According to the results of our study, residual infection was identified as the most important source of bTB breakdowns in Italy, followed by pasture sharing and interaction with wildlife. This finding aligns with similar studies performed in Spain by Guta et al. (32), and more recently by Ciaravino et al. (43), which reported residual infection as the primary source of bTB breakdown. The percentage of recurrent breakdowns in our study (11%) was lower than values obtained in studies conducted in other European countries where bTB is still present. In Great Britain, Karolemeas et al. (44) reported that 23% of breakdowns recurred within 12 months of the previous breakdown ending and around 38% within 24 months. In Spain, Guta et al. (32) estimated a 22% recurrence of bTB breakdowns in 2009–2011, while Ciaravino et al. (43) reported a 36% recurrence a few years later. Moreover, a history of bTB has been evidenced as a robust predictor of future breakdown risk in the United Kingdom and Ireland (8, 11). The presence of false-negative animals due to the skin test’s failure to detect all infected animals or suboptimal veterinary practices could be regarded as an important reason to explain the large number of breakdowns attributed to residual infection (6, 7). Probably, our results are affected by the fact that, especially in DFS provinces, all animals of infected herds, including test-negative animals, are often slaughtered. This measure certainly reduces the risk of MTBC persistence on farms, potentially contributing to the lower recurrence rate observed in our study.

Sharing of pasture and interactions with wildlife were identified as the second and third sources of bTB, respectively, similar to findings in Spain (32, 43). Farms with large pasture areas and bTB-infected neighbors have a higher risk of bTB infection in Mediterranean countries (12, 45). In high-prevalence areas where pasture is extensively used with poor biosecurity measures, it is challenging to distinguish whether the MTBC infection originates from wildlife or domestic animals sharing the same pasture. Moreover, in both central and southern Italy, as well as in central and southern Spain, high bTB prevalence has been detected in wild boar, suggesting that this population could be an important source of infection for cattle (15–17, 26). Nevertheless, the role of wildlife in bTB transmission could be overestimated, as our spatial data on bTB presence in wildlife are available only at the regional level, rather than at the more precise provincial or municipal level. In the north of the country, the prevalence of infected wildlife reservoirs seems to be lower, suggesting that their role as bTB reservoirs is probably less significant (46). Nevertheless, in a DFS region of central Italy, wildlife has been recently proposed as a potential source of infection, alongside farm-to-farm transmission due to proximity to other infected farms (47). However, the northern regions have a higher concentration of dairy herds with intensive production systems and minimal pasture use compared to the rest of the country. For these reasons, in our study, the sharing of pastures and interaction with wildlife did not emerge as probable sources of breakdowns in dairy herds.

Differences in bTB breakdown sources were also observed based on the disease status of the province where the herd was located. In DFS provinces, residual infection and the introduction of infected animals were the most frequent sources of bTB breakdowns. Herds may also become infected due to external sources. In this study, the importance attributed to the introduction of infected cattle was found to be less significant than in previous studies. In Northeast England, Gopal et al. (10) identified the purchase of infected cattle as the most likely source of infection in 30 out of 31 bTB breakdowns, following restocking from high-risk areas following the 2001 foot and mouth disease epidemic. However, it is important to consider that in Italy, pre- and post-movement tests are systematically carried out in non-DFS areas. This measure helps decrease the risk of introducing infected animals, as highlighted in Scotland and England, where the introduction of these control measures has reduced the risk of infection (48). In the United Kingdom, the purchase of animals from high-risk areas has been identified as a risk factor for low-prevalence areas (48, 49). Our findings align with these observations, as in DFS provinces, the introduction of animals was the second source of breakdowns. Additionally, the illegal introduction of animals should also be considered. In Italy, animals in non-DFS provinces are identified with both an ear tag and electronically with a ruminal bolus, whereas in the rest of the country, two ear tags are generally used. Animals must be identified within 20 days of age, but for those born on pasture, identification can be postponed up to 6 months by derogation. For this reason, in our decision tree for animal introduction, we included the presence of an excessive number of twins (>20% compared to the number of cows, considering a physiological percentage of twin births of 5% (50)) as a proxy for the illegal introduction of calves that presumably came from unidentified herds under animal movement restrictions.

We decided to consider a source of herd breakdown only if the likelihood of occurrence was at least “likely,” corresponding to an epidemiological link with another breakdown or the detection of the same genotype within the same municipality of the farm or pasture. Based on this threshold, 71% of the studied herds (248 out of 348) were classified as having an unknown source of breakdown. The remaining “unknown” cases (10 out of 348) corresponded to breakdowns with more than two probable sources. The 74% of breakdowns with an “unknown” source of infection is a worse result compared to those reported in Spain, Great Britain, and Northern Ireland, where the source of infection could not be identified in 42, 40, and 32% of cases, respectively (32, 51, 52). The proportion of breakdowns with an unknown source decreases to 51% (156 out of 305) when considering the opinions of veterinarians. However, as in similar studies conducted in Spain (32, 43), the comparison between results based on decision trees and conclusions of the veterinary officers showed very poor agreement. Both methods, the decision trees and the opinions of veterinary officers, have their strengths and weaknesses, but they are partially based on different types of information and should be considered complementary. The decision trees follow standardized procedures based on expert opinion and literature review. Moreover, they also use data such as laboratory data and the list of bTB breakdowns, which are often unavailable to veterinary officers during official investigations. Genotype data play a crucial role in epidemiological investigations, providing valuable insights into transmission dynamics; however, the timely submission of isolates for genotyping is often a challenge, leading to delays in obtaining genotype results, which are sometimes available only after a significant lag. Our decision trees have some limitations related to the fixed nature of the questions. They were basically designed for surveillance systems based on annual herd checks, meaning that the time frames used (e.g., 2 years for purchased cattle) may be too stringent for areas with less frequent herd control, such as DFS zones of Italy, where checks may occur every 3–5 years. In addition, the decision trees do not account for specific management practices, facilities, or the role of the herd owner in each infected herd. Besides, the veterinary officers had direct field experience and personal interaction with farm owners, allowing them to gather first-hand information. In our study, the same criteria were applied to all the herds, whereas veterinary officers’ assessments may have shown higher heterogeneity due to different regional or individual knowledge and perceptions of the risk associated with different epidemiological scenarios and infection patterns. A significant discrepancy was observed between the decision trees outputs and the source attribution assessments made by veterinary officers, particularly in relation to transmission from neighboring farms (not explicitly listed as a source of infection in the SIMAN questionnaire) and interactions with wildlife reservoirs.

In conclusion, this study provided an overview of bTB breakdowns in Italy and investigated the probable sources of infection using decision trees. These tools allowed us to identify a likely source of infection in about 26% of breakdowns. The results of these investigations, although based on scientific criteria, are substantially at odds with the conclusions of the veterinary officers who carried out the breakdown investigations. It would be desirable to harmonize the criteria used in the epidemiological investigations conducted by veterinary officers, including the use of decision trees. These tools, added to the “classic” methodologies of inquiry, should lead to improved effectiveness in identifying sources of infection in bTB breakdowns in Italy.

## Data Availability

The datasets presented in this study can be found in online repositories. The names of the repository/repositories and accession number(s) can be found below: doi: 10.5281/zenodo.15187345.

## Footnotes

^1^ https://www.vetinfo.it/

^2^ https://www.mbovis.org/
