# Supplementary material for: Bovine tuberculosis source attribution using decision tree analysis: breakdown investigations in Italy (2022–2023)
Source: Front Vet Sci. 2025 Aug 13;12:1609526. doi: 10.3389/fvets.2025.1609526 (PMC12380583; doi:10.3389/fvets.2025.1609526)
Supplement: Supplementary file 1 [file Table_1.DOCX]

### Bovine Tuberculosis source attribution using decision tree analysis: Breakdown Investigations in Italy (2022-2023)

Marco Tamba^1*^, Giorgio Galletti^1^, Daniela Loda^2^, Sara Salvato^1^, Marco De Nardi^3^, Maria Beatrice Boniotti^2^

^1^Epidemiology Unit, Sanitary Directorate, Istituto Zooprofilattico Sperimentale della Lombardia e dell’Emilia Romagna “B. Ubertini”, Bologna, Italy

^2^National Reference Laboratory for Bovine Tuberculosis, Department of Animal Health, Istituto Zooprofilattico Sperimentale della Lombardia e dell’Emilia Romagna “B. Ubertini”, Brescia, Italy

^3^Department of Veterinary Medical Sciences (DIMEVET), Alma Mater Studiorum - University of Bologna, Ozzano dell'Emilia (BO), Italy

Supplementary Material

**Figure S1: Decision tree diagram for residual infection**


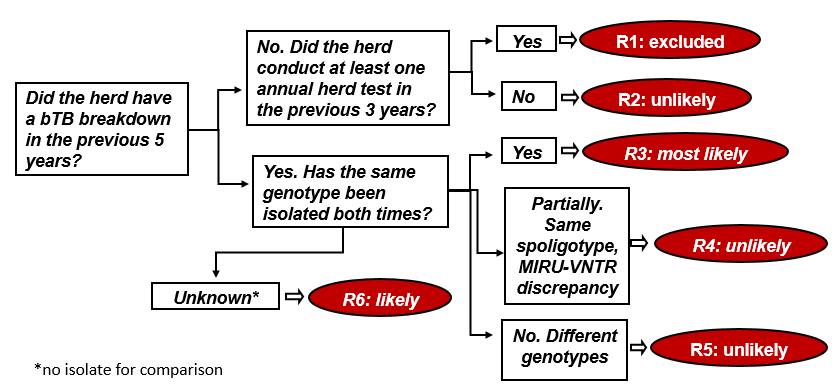


# Table S1: Frequencies of possible events for residual infection in bTB breakdowns. Italy, 2022-2023

| **L1** | **L2** | **L3** | **N** | **%** | **Event code** | **probability of MTBC entry** |
| --- | --- | --- | --- | --- | --- | --- |
| Did the herd have a bTB breakdown in the previous 5 years? | No. Did the herd conduct at least one annual herd test in the previous 3 years? | Yes | 274 | 78.7% | R1 | excluded |
|  |  | No | 34 | 9.8% | R2 | unlikely |
|  | Yes. Has the same genotype been isolated both times? | Yes | 10 | 2.9% | R3 | most likely |
|  |  | Partially. Same spoligotype, MIRU-VNTR discrepancy | 0 | 0.0% | R4 | unlikely |
|  |  | No. Different genotypes | 1 | 0.3% | R5 | unlikely |
|  |  | Unknown* | 29 | 8.3% | R6 | likely |
| **Total** |  |  | **348** | **100.0%** |  |  |

*No isolate for comparison

**Figure S2: Decision tree diagram for introduction of infected cattle from other herds**


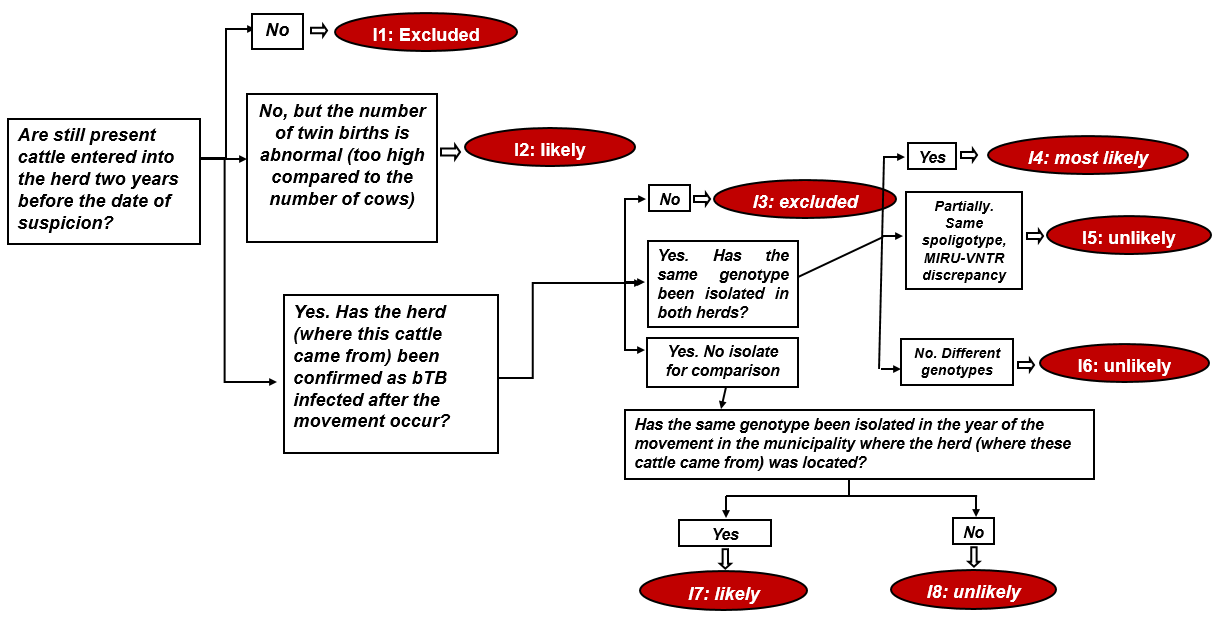


# Table S2: Frequencies of possible events for introduction of infected cattle from other herds in bTB breakdowns. Italy, 2022-2023

| **L1** | **L2** | **L3** | **L4** | **L5** | **N** | **%** | **Event code** | **probability of MTBC entry** |
| --- | --- | --- | --- | --- | --- | --- | --- | --- |
| Are still present cattle entered into the herd two years before the date of suspicion? | No |  |  |  | 109 | 31.3% | I1 | excluded |
|  | No. The number of twin births is abnormal (too high compared to the number of cows) |  |  |  | 4 | 1.1% | I2 | likely |
|  | Yes. Has the herd (where this cattle came from) been confirmed as bTB infected after the movement occur? | No |  |  | 193 | 55.5% | I3 | excluded |
|  |  | Yes. Has the same genotype been isolated in both herds? | Yes |  | 14 | 4.0% | I4 | most likely |
|  |  |  | Partially. Same spoligotype, MIRU-VNTR discrepancy |  | 5 | 1.4% | I5 | unlikely |
|  |  |  | No. Different genotypes |  | 7 | 2.0% | I6 | unlikely |
|  |  | Yes. No isolate for comparison | Has the same genotype been isolated in the year of the movement in the municipality where the herd (where these cattle came from) was located? | Yes | 1 | 0.3% | I7 | likely |
|  |  |  |  | No | 15 | 4.3% | I8 | unlikely |
| **Total** |  |  |  |  | **348** | **100.0%** |  |  |

**Figure S3: Decision tree diagram for sharing of pastures with infected herds**


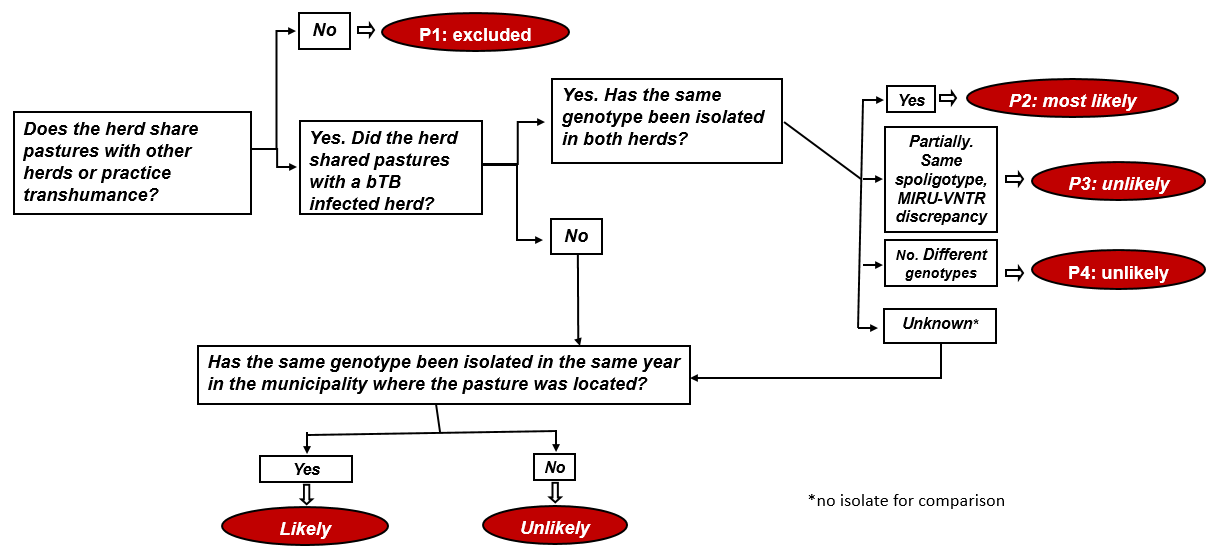


# Table S3: Frequencies of possible events for sharing of pastures with infected herds in bTB breakdowns. Italy, 2022-2023

| **L1** | **L2** | **L3** | **L4** | **L5** | **N** | **%** | **Event code** | **probability of MTBC entry** |
| --- | --- | --- | --- | --- | --- | --- | --- | --- |
| Does the herd share pastures with other herds or practice trans-humance? | No |  |  |  | 163 | 46.8% | P1 | excluded |
|  | Yes. Did the herd shared pastures with a bTB infected herd? | Yes. Has the same genotype been isolated in both herds? | Yes |  | 12 | 3.4% | P2 | most likely |
|  |  |  | Partially. Same spoligotype, MIRU-VNTR discrepancy |  | 0 | 0.0% | P3 | unlikely |
|  |  |  | No. Different genotypes |  | 2 | 0.6% | P4 | unlikely |
|  |  | No/Unknown (No isolate for comparison) | Has the same genotype been isolated in the same year in the municipality where the pasture was located? | Yes | 26 | 7.5% | P5 | likely |
|  |  |  |  | No | 145 | 41.7% | P6 | unlikely |
| **Total** |  |  |  |  | **348** | **100.0%** |  |  |

**Figure S4: Decision tree diagram for contiguous spread from infected neighboring herds**


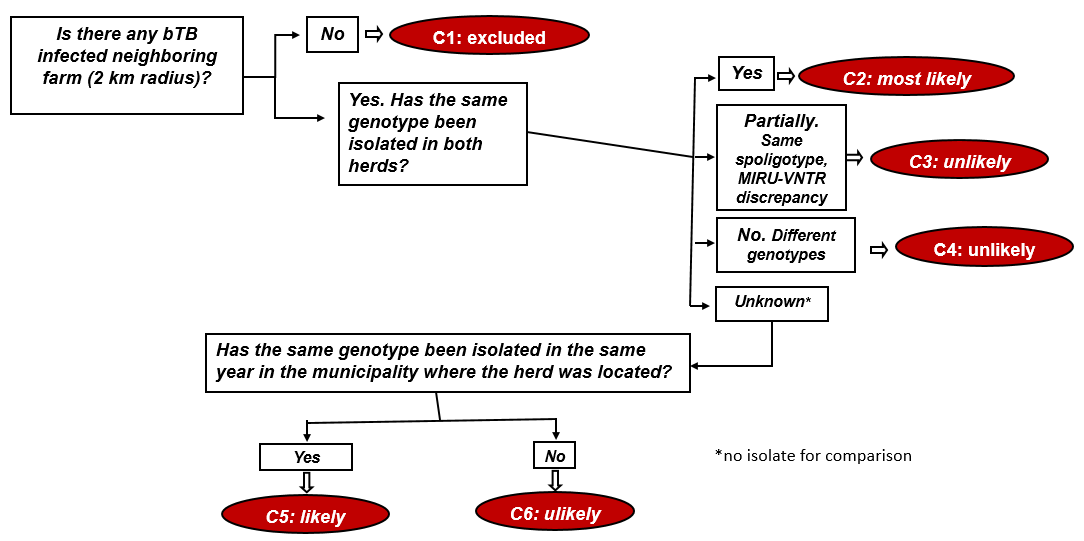


# Table S4: Frequencies of possible events for contiguous spread from infected neighboring herds in bTB breakdowns. Italy, 2022-2023

| **L1** | **L2** | **L3** | **L4** | **L5** | **N** | **%** | **Event code** | **probability of MTBC entry** |
| --- | --- | --- | --- | --- | --- | --- | --- | --- |
| Is there any bTB infected neighboring farm (2 km radius)? | No |  |  |  | 293 | 84.2% | C1 | excluded |
|  | Yes. Has the same genotype been isolated in both herds? | Yes |  |  | 12 | 3.4% | C2 | most likely |
|  |  | Partially. Same spoligotype, MIRU-VNTR discrepancy |  |  | 6 | 1.7% | C3 | unlikely |
|  |  | No. Different genotypes |  |  | 6 | 1.7% | C4 | unlikely |
|  |  | Unknown (No isolate for comparison) | Has the same genotype been isolated in the same year in the municipality where the herd was located? | Yes | 2 | 0.6% | C5 | likely |
|  |  |  |  | No | 29 | 8.3% | C6 | unlikely |
| **Total** |  |  |  |  | **348** | **100.0%** |  |  |

**Figure S5: Decision tree diagram for interaction with wildlife reservoirs at pasture**


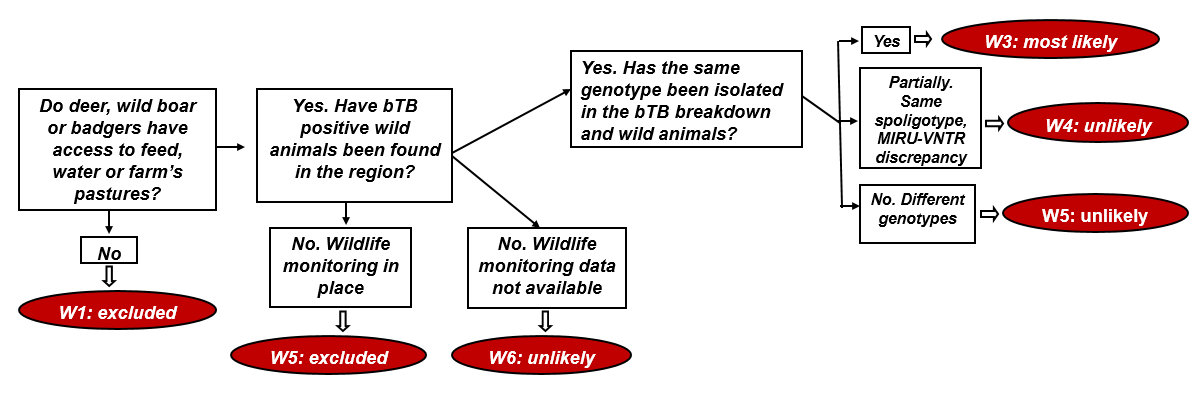


# Table S5: Frequencies of possible events for interaction with wildlife reservoirs at pasture in bTB breakdowns. Italy, 2022-2023

| **L1** | **L2** | **L3** | **L4** | **N** | **%** | **Event code** | **probability of MTBC entry** |
| --- | --- | --- | --- | --- | --- | --- | --- |
| Do deer, wild boar or badgers have access to feed, water or farm’s pastures? | No |  |  | 157 | 45.1% | W1 | excluded |
|  | Yes. Have bTB positive wild animals been found in the region? | Yes. Has the same genotype been isolated in the bTB breakdown and wild animals? | Yes | 25 | 7.2% | W2 | most likely |
|  |  |  | Partially. Same spoligotype, MIRU-VNTR discrepancy | 31 | 8.9% | W3 | unlikely |
|  |  |  | No. Different genotypes | 36 | 10.3% | W4 | unlikely |
|  |  | No. Wildlife monitoring in place |  | 89 | 25.6% | W5 | excluded |
|  |  | No. Wildlife monitoring data not available |  | 10 | 2.9% | W6 | unlikely |
| **Total** |  |  |  | **348** | **100.0%** |  |  |
